# S6K2-mediated regulation of TRBP as a determinant of miRNA expression in human primary lymphatic endothelial cells

**DOI:** 10.1093/nar/gkw631

**Published:** 2016-07-12

**Authors:** Matthew J. Warner, Katherine S. Bridge, James P. Hewitson, Michael R. Hodgkinson, Alex Heyam, Bailey C. Massa, Jessica C. Haslam, Maria Chatzifrangkeskou, Gareth J.O. Evans, Michael J. Plevin, Tyson V. Sharp, Dimitris Lagos

**Affiliations:** 1 Centre for Immunology and Infection, Department of Biology and Hull York Medical School, University of York, Wentworth Way, York, YO10 5DD, UK; 2 Centre of Molecular Oncology, Barts Cancer Institute, John Vane Science Centre, Charterhouse Square, Queen Mary University London, London, EC1M 6BQ, UK; 3 Department of Biology, University of York, Wentworth Way, York, YO10 5DD, UK

## Abstract

MicroRNAs (miRNAs) are short non-coding RNAs that silence mRNAs. They are generated following transcription and cleavage by the DROSHA/DGCR8 and DICER/TRBP/PACT complexes. Although it is known that components of the miRNA biogenesis machinery can be phosphorylated, it remains poorly understood how these events become engaged during physiological cellular activation. We demonstrate that S6 kinases can phosphorylate the extended C-terminal domain of TRBP and interact with TRBP *in situ* in primary cells. TRBP serines 283/286 are essential for S6K-mediated TRBP phosphorylation, optimal expression of TRBP, and the S6K-TRBP interaction in human primary cells. We demonstrate the functional relevance of this interaction in primary human dermal lymphatic endothelial cells (HDLECs). Angiopoietin-1 (ANG1) can augment miRNA biogenesis in HDLECs through enhancing TRBP phosphorylation and expression in an S6K2-dependent manner. We propose that the S6K2/TRBP node controls miRNA biogenesis in HDLECs and provides a molecular link between the mTOR pathway and the miRNA biogenesis machinery.

## INTRODUCTION

Following their generation from the DROSHA and DICER complexes (1), mature miRNAs associate with members of the Argonaute (AGO) and TNRC6 (trinucleotide repeat containing 6) families of proteins, leading to the formation of the RNA-induced silencing complex (RISC). MiRNAs play a central role in mammalian development (2). This is evidenced by the fact that genetic deletion of DICER in mice causes vascular abnormalities leading to embryonic lethality (3). Similarly, endothelial-specific deletion of DICER (4,5), DROSHA (6) or AGO2 (7) impairs cell survival and responses to angiogenic stimuli. Although the requirement for the miRNA biogenesis machinery in mammalian cell development and function has been extensively demonstrated, a question that is significantly less explored is whether, and to what extent, regulation of the miRNA processing machinery occurs during human primary cell activation. Additionally, the intracellular signalling mechanisms leading to post-translational modification of the miRNA biogenesis machinery in non-transformed cells, including DROSHA, DGCR8 (DiGeorge syndrome chromosomal region 8), TRBP (TARBP2; transactivating response RNA (HIV-1)-binding protein 2) and AGO2 (8–11), remain poorly understood. For example, it is thought that TRBP assists DICER-mediated precursor miRNA processing (12,13), it controls mature miRNA length and strand selection (14), and that it is a component of the RISC loading complex (15). TRBP has three domains, the first two of which bind dsRNA, whilst the C-terminal domain is thought to mediate interactions with DICER and other proteins (16–18). Paroo and colleagues demonstrated that TRBP has two phosphorylation sites in the linker domain between the first two dsRBD domains (serines 142, 152) and two at the extended third domain (serines 283 and 286) (10). Furthermore, Kim *et al.* identified 10 more potential TRBP phosphorylation sites (19). Both studies suggested that TRBP hyper-phosphorylation controls its stability (10,19). However, the relevance of TRBP post-translational modifications to its function in physiological responses in human primary cells remains poorly understood.

Here, we identify a novel mechanism controlling miRNA biogenesis through S6 kinase (S6K)-mediated phosphorylation of TRBP. We show that S6 kinases phosphorylate the extended C terminal region of TRBP (TRBP-D3). TRBP serines 283/286 are required for *in vitro* TRBP-D3 phosphorylation, serine 283 being the predominant S6 kinase target site. In parallel, serines 283/286 are crucial for optimal TRBP expression in human primary cells. We show that endogenous S6K1 or S6K2 interact *in situ* with TRBP in primary HDLECs. Using the ANG/TIE2 signalling pathway (20) in primary HDLECs as a model of physiological S6K activation, we show that TRBP phosphorylation and expression are regulated by S6K2. The ANG/TIE2 system is essential in embryonic vasculature development and postnatal angiogenesis, and its deregulation is associated with pathological conditions such as cancer, chronic inflammation, and cardiovascular disease (20). Functionally, we show that S6K2 contributes to ANG1-mediated TRBP activation that results in enhanced expression of several highly expressed HDLEC miRNAs. Our findings reveal a previously unknown molecular mechanism governing post-translational control of the miRNA biogenesis machinery in human primary cells.

## MATERIALS AND METHODS

### Cell culture

HDLEC were purchased from Promocell and grown in endothelial growth MV (microvascular) medium (Promocell, catalogue number C-22020) supplemented with 10 ng/ml VEGF-C (R&D systems; Full Media). As Basal media we used the endothelial growth MV medium without any supplements (Promocell, catalogue number C-22220). All experiments were performed before passage 6. HDLECs were seeded in either six-well plates at 8 × 10^4^ cells per well, or in 12-well plates at 1.5 × 10^4^ cells per well and all ANG1 and ANG2 (R&D Systems) treatments were performed at 300 ng/ml for varying times, ranging from 30 min to 24 h. HEK 293 cells were grown in Dulbecco's modifiedEagle's medium (DMEM), 10% Fetal Calf Serum (FCS).

### siRNA transfection of primary cells

ON-TARGETplus small interfering RNA (siRNA) oligonucleotides were purchased from Dharmacon. HDLECs were transfected with a total of 100 nM siRNA using TransIT-siQUEST transfection reagent (Mirus) and Opti-MEM medium (Gibco) for 6 h before being replaced with full endothelial growth medium. After 24 h, HDLECs were starved for 16 h before pharmacological pre-treatment and/or ANG1 stimulation.

### Quantitative RT-PCR (qRT-PCR)

HDLEC total RNA was extracted using Qiazol and miRNeasy RNA extraction kits (Qiagen). cDNA synthesis of mature miRNAs was performed using Taqman miRNA reverse transcription kits (Applied Biosystems) and mRNA cDNA synthesis was completed using the Superscript III reverse transcriptase (Invitrogen). qRT-PCR quantification of DROSHA, DICER, AGO2 and PACT was performed using Taqman gene expression assays and all mature or primary miRNAs examined were detected using Taqman miRNA assays (Applied Biosystems). Pri-miR-126, TRBP and GAPDH were quantified using the following optimized forward and reverse primers by SYBR Green qRT-PCR (Applied Biosystems):

pri-miR-126_forward: 5′-TATCAGCCAAGAAGGCAGAA-3′
pri-miR-126_reverse: 5′- CGTGGCGTCTTCCAGAAT-3′
TRBP_forward: 5′- GGGAAGACGCCTGTGTACGA-3′
TRBP_reverse: 5′- GGTGACCCGGAAGGTGAAA-3′
GAPDH_forward: 5′-GGAGTCAACGGATTTGGTCGTA-3′
GAPDH_reverse: 5′-GGCAACAATATCCACTTTACCAGAGT-3′

Relative mRNA levels were calculated using the ΔΔCT method. mRNA and primary miRNA levels were normalized to GAPDH, and mature miRNA levels were normalized to U6 RNA. Absolute miRNA copy number estimations were performed using standard curves constructed following reverse transcription of known quantities of synthetic RNA oligonucleotides (Sigma) corresponding to the mature sequence of miR-126 and miR-16.

### Proximity ligation assay

Subconfluent HDLECs were fixed in 4%  (v/v) paraformaldehyde in PBS, permeabilized in 0.3% Triton and blocked in 2% (w/v) BSA/PBS 0.05% Tween for 1 h. Primary antibodies [TRBP (Abcam, ab42018) 2.5 μg/ml, S6K2 (Abnova, B02P), S6K1 (Abcam, ab119252), 2.5 μg/ml, IgG Mouse (Cell Signaling) 12.5 μg/ml, IgG Rabbit (Cell Signaling) 2.5 μg/ml and Dicer (Abcam) 20 μg/ml)] were incubated overnight at 4°C in blocking buffer. Washes were performed in blocking buffer, followed by a final wash in 10% blocking buffer/PBS. PLA was performed using Duolink, as per the manufacturer's instructions (Olink Biosciences, Sigma, DUO92102). Cells were stained for F-actin with Alexafluor 488 Phalloidin (Life Technologies, A12379) 1:40 (v/v) in blocking buffer for 20 min, and then mounted in DAPI-containing mounting media, for nuclear staining (Sigma, DUO82040). Images were taken using a Zeiss LSM 510 inverted confocal microscope and the number of PLA events per cell in 100 cells per condition was counted.

### Recombinant protein expression

Recombinant TRBP-D1/2 (residues 19–228) was generated and purified as previously reported (21). TRBP-D3 (residues 258–366), TRBP-D3(DD) (258–366; S283D, S286D) or PACT-D3 (208–313) were over expressed in *Escherichia coli* BL21(DE3) as fusion proteins and purified by immobilized metal affinity chromatography (IMAC). The eluted fusion protein was subjected to protease cleavage and the protein of interest separated from the fusion protein tag by a second IMAC step. All protein samples were then further purified by size exclusion chromatography in 20 mM Tris pH 7.5, 200 mM NaCl, 1 mM DTT. Fractions containing TRBP-D3, TRBP-D3(DD) or PACT-D3 were identified by SDS-PAGE and concentrated to 2 mg/ml.

### *In vitro* kinase assay

*In vitro* phosphorylation of recombinant proteins were performed at 30°C in a volume of 75 μl in kinase reaction buffer (25 mM Tris–HCl, pH 7.5, 0.01% Triton, 0.05 mM EGTA, 0.5 mM sodium orthovanadate, 5 mM β-glycerophosphate, 2.5 mM DTT and 10 mM MgCl_2_), with 160 nM active recombinant S6K1, S6K2, AKT1 or ERK2 (Invitrogen) and 66 μM TRBP-D3, TRBP-D3(DD), TRBP-D1/D2 or PACT-D3. Reactions were initiated by the addition of 20 μCi [γ-32P]ATP and unlabelled ATP to a final concentration of 250 μM. Aliquots of 15 μl were removed at 0, 1, 2, 3 and 4 h and the reactions terminated by the addition of 2× sodium dodecylsulphate (SDS) sample buffer. Incorporation of ^32^P was quantified by resolving the reactions on 15% sodium dodecylsulphate-polyacrylamide gel electrophoresis (SDS-PAGE) gels, staining the gel with Coomassie, excising the bands corresponding to the substrates and then subjecting them to liquid scintillation counting. To normalise the ^32^P incorporation to protein content, the intensity of Coomassie staining for each substrate band was determined by densitometry.

### Mass spectrometry

Following *in vitro* phosphorylation, the reactions were quenched by the addition of 50 μl SDS loading buffer. Samples were run on a 15% SDS-PAGE gel, 20 μl per lane, and stained with Coomassie. Protein bands were excised from the gel, destained, reduced, alkylated and digested with trypsin following standard protocols. The resulting peptide solutions were enriched for phosphopeptides using TiO2 micro-columns. Following enrichment, samples were spotted directly onto the MALDI target plate in DHB matrix (20 mg/ml 2,5-dihydroxybenzoic acid, 1% phosphoric acid). MS/MS analysis was performed on a Bruker Ultraflex in positive ion, reflector mode. MS spectra were acquired in the mass range *m*/*z* 800–4000. Peaks of interest on the MS spectra for each sample were then selected for MS/MS analysis. The MS/MS data was submitted to database searching against an in-house database, using a locally running copy of the Mascot software (Matrix Science) through a Biotools (Bruker Daltonics) interface.

### Western blotting and co-immunoprecipitation assays

HDLEC protein was extracted using RIPA buffer: 150 mM NaCl; 10 mM Tris–HCl pH 7.2; 0.1% SDS; 0.1% Triton X-100; 1% sodium deoxycholate; 5 mM EDTA; 1% protease (P8340) and phosphatase inhibitors 2 (P5726) and 3 (P0044) cocktails (all inhibitor cocktails purchased from Sigma). Equal total amounts of protein were resolved on polyacrylamide gels. Antibodies against TIE2 (#4224, AB33), phospho (#4370, D13.14.4E) and total ERK (#4695, 137F5), phospho (Ser473, #4060, D9E; Ser308, #2965, C31E5) and total AKT (#4691, C67E7), phospho (#2974) and total mTOR (#2983, 7C10), phospho-S6K1 (#9234, 108D2) and total S6K1 (#2708, 49D7), total S6K2 (#14130), DICER (#5362, D38E7), DROSHA (#3364, D28B1), phospho (#4858, D57.2.2E) and total S6RP (#2217, 5G10) and AGO (#2897, C34C6) were purchased from Cell Signaling Technology. Antibodies to PACT (PRKRA) (ab31967) and β-actin (ab6276) were purchased by Abcam. Antibodies to TRBP were purchased from Abcam (ab42018) and Proteintech (15753-1-AP). Reactivity was detected with HRP-conjugated secondary antibodies and quantified by ECL (GE Healthcare) or Clarity ECL (Bio-Rad). Densitometry was performed using ImageJ software.

For co-immunoprecipitation of ectopically expressed proteins, subconfluent HEK293 cells were lysed by the addition of ice-cold lysis buffer (Cell Signaling Technology, #9803, 20 mM Tris (pH 7.5), 150 mM NaCl, 1 mM EDTA, 1 mM EGTA, 1% Triton X-100, 2.5 mM sodium pyrophosphate, 1 mM β-glycerophosphate, 1 mM Na3VO4, 1 μg/ml Leupeptin, adding 1 mM PMSF immediately prior to use). Lysates were incubated for 10 min on ice and centrifuged at 13 000  rpm for 10 min. The lysate was then pre-cleared with rabbit IgG (Cell Signaling Technology, #2729) for 2 h at 4°C followed by incubation with protein A beads (#8687) for 20 min at room temperature. The cleared lysates were incubated with 1  μg anti-S6K2 (#14130) or normal IgG (#2729) for 16 h at 4°C. The immune complexes were incubated with protein A beads (#8687) for 20 min, washed 5 times, and eluted in 40 μl 4× SDS-PAGE sample buffer.

### Phosphatase treatment of cell lysates

Protein extracts were prepared as before, except cells were lysed in modified RIPA buffer lacking EDTA, but supplemented with protease and phosphatase inhibitor cocktails (for control samples) or protease inhibitor cocktail alone (for samples to be phosphatase treated). For λ phosphatase treatment, soluble protein lysates (10 μg) were incubated with or without λ phosphatase (400 U; NEB) for 45 min at 30°C.

### Lentiviral cloning and transduction of HDLECs

TRBP (TARBP2, NCBI Reference Sequence NM_134323.1) amplified from HDLEC cDNA and subcloned into the pSIN lentiviral vector using the NotI and BamHI restriction enzymes and the following primers:

Forward: TRBP_BamH1 = 5′-cgcggatccATGAGTGAAGAGGAGCAAGGC-3′
Reverse TRBP_Not1 = 5′-aaggaaaaaagcggccgcCAGCTGGGGCTTCACTTG-3′

For lentiviral transduction, virions were produced in HEK293T cells as described previously (22). Site-directed mutagenesis was performed using the QuickChange Stratagene kit following manufacturers’ instructions. HDLECs were infected with TRBP-WT, TRBP-AA, TRBP-DD lentiviruses 72 h before analysis. For HEK293 infections, 10^6^ cells were infected with 1–2 ml of indicated lentiviruses and cells were grown until 80% confluency before being harvested for immunoprecipitation experiments.

### MicroRNA profiling of HDLEC

**Figure 1.**
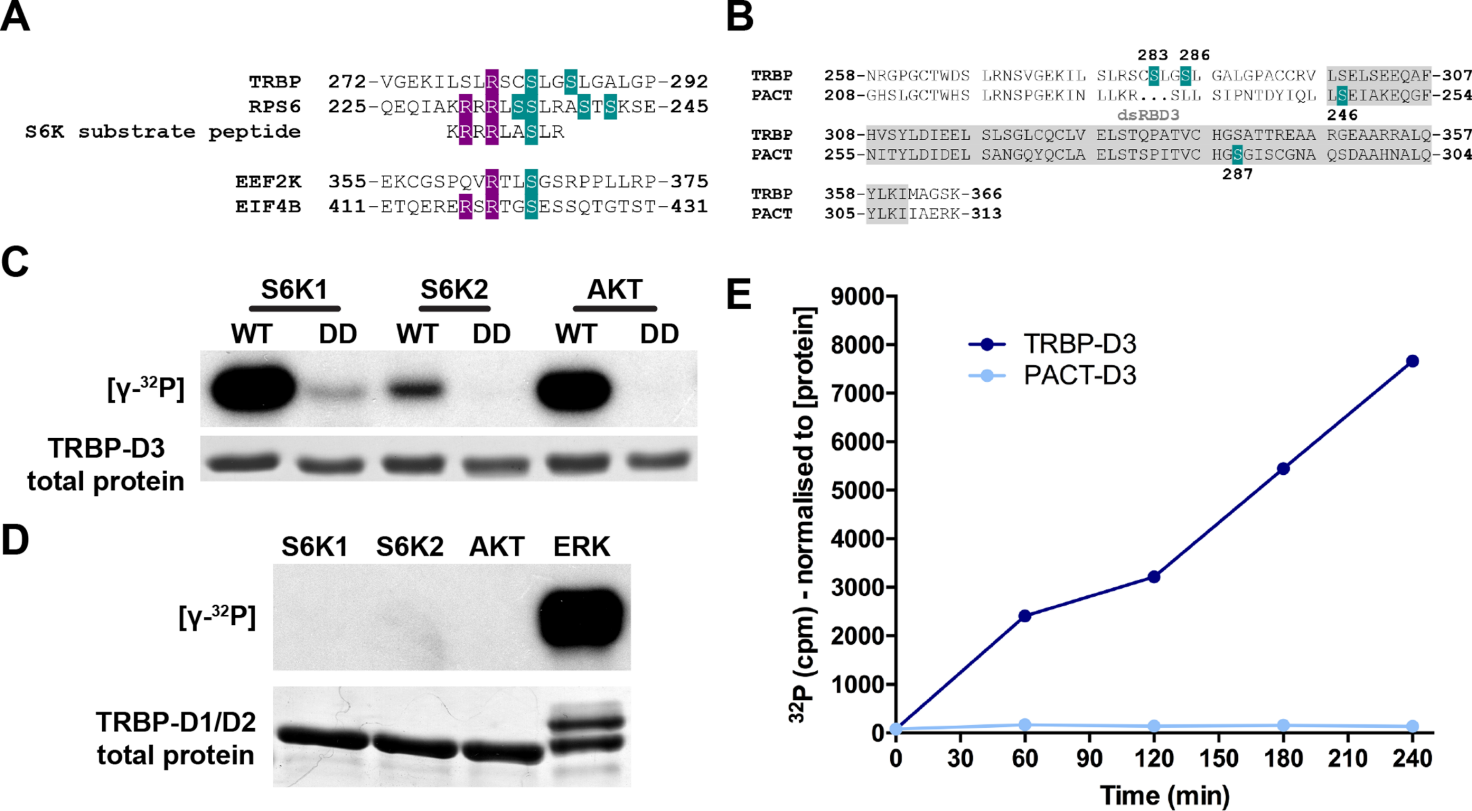
TRBP-D3 is a substrate of S6 kinases. (**A**) Alignment of candidate S6K phosphorylation sites on TRBP and known sites on S6RP (shown in green). A model S6K substrate peptide is also shown for reference. Two validated S6K substrates containing the RXRXXS or RXXS consensus motifs (R in purple) are also shown. (**B**) TRBP-D3 (aa258-366) and PACT-D3 (aa208–313) sequence alignment, illustrating known TRBP and PACT phosphorylation sites (green). (**C**) [γ-32P] autoradiogram and total protein (detected by Coomassie staining) after kinase assay incubation (4 h) of TRBP-D3 and TRBP-D3(DD) with active S6K1, S6K2, or AKT1. (**D**) [γ-32P] autoradiogram and total protein (detected by Coomassie staining) after kinase assay incubation (4 h) of TRBP-D1/D2 with recombinant S6K1, S6K2, AKT1 or ERK2. A band shift due to ERK phosphorylation is observed for TRBP-D1/D2. (**E**) [γ-32P] counts for TRBP-D3 and PACT-D3 during 4 h *in vitro* phosphorylation by recombinant S6K2.

MicroRNA profiling was performed using Agilent miRNA chips v19.0, containing 60,180 probes for 2006 miRNAs. Four replicates of ANG1-treated and control HDLECs were compared after 24 h treatment. Following quality control, raw data import and normalization together with differential expression analysis were performed using the R Bioconductor software package limma (23). The signal intensity of each probe on each array was quantified by taking the median foreground signal with no background correction. Subsequently all arrays were filtered to remove control probes and were normalized using the quantile method, which sets intensities to have the same empirical distribution across arrays. The differential analysis was performed using a moderated t-statistic computed for each contrast for each probe. This analysis was performed on the whole dataset taking the mapping into account as previously described (23). In Figure 1B, detectable miRNAs were identified using an arbitrary cut-off point of 6.4 for normalized signal (Supplementary Table S1). This cut-off point allows inclusion of lowly expressed (50–100 copies per cell) but detectable miRNAs (e.g. miR-132 (24)).

### Statistical analysis

All experiments were performed as independent replicates (*n* ≥ 3) and are illustrated as means including error bars corresponding to standard deviation from the mean (SD). *P*-values were calculated using Student's *t*-test when only comparing to a negative control sample, one-way ANOVA when performing multiple comparisons between conditions (one class), and two-way ANOVA when performing multiple comparisons between different conditions (two classes, e.g. Figure 7D). The statistical significance of ANG1-induced miRNA expression enhancement (Figure 7A) was calculated using a chi-square test against the null hypothesis that there was no bias towards up- or down-regulation (i.e. half the miRNAs show a positive FC and the half negative FC). A *P*-value <0.05 was considered significant.

## RESULTS

### TRBP serines 283/286 are required for optimal TRBP expression in human primary cells and *in vitro* phosphorylation of TRBP-D3 by S6 kinases

We observed that two previously identified (10) TRBP phosphorylation sites (S283, S286) in the extended third, non-canonical dsRBD of TRBP (TRBP-D3) displayed features of potential S6 kinase substrate motifs (RXXS, Figure 1A) (25,26). This motif is not observed for any of the other twelve candidate TRBP phoshorylation sites (10,19), nor in the equivalent domain of PACT (PACT-D3), a TRBP homologue and DICER co-factor with two known phosphorylation sites (27,28) in its third domain (Figure 1B). As S6 kinases play a central role in eukaryotic translation we pursued this potential link to miRNA biogenesis further. *In vitro* kinase assays demonstrated that TRBP-D3 can be phosphorylated by S6K1, S6K2, but also AKT1 (Figure 1C), inferring that, at least *in vitro*, this domain can be an AGC kinase substrate (25,26). S6K2 and AKT1 had nearly undetectable kinase activity when a TRBP mutant (TRBP-D3(DD), in which the two candidate serines were mutated to aspartate and thus could not be phosphorylated, was used as a substrate. S6K1 demonstrated some residual activity against TRBP-D3(DD) possibly due to other serines present in the domain. No S6K-mediated phosphorylation was observed when using TRBP-D1/2, a construct containing the two N-terminal dsRBDs of TRBP (Figure 1D). ERK2 was used as a control and was able to phosphorylate TRBP-D1/2 as reported previously (10). Similarly, S6K2 did not show any detectable reactivity with PACT-D3 (Figure 1E). Consistent with the observed AGC kinase binding motif and the above results, mass spectrometry analysis of *in vitro* phosphorylated TRBP-D3 confirmed that at least serine 283 is directly phosphorylated by AGC kinases (Supplementary Figure S1A).

**Figure 2.**
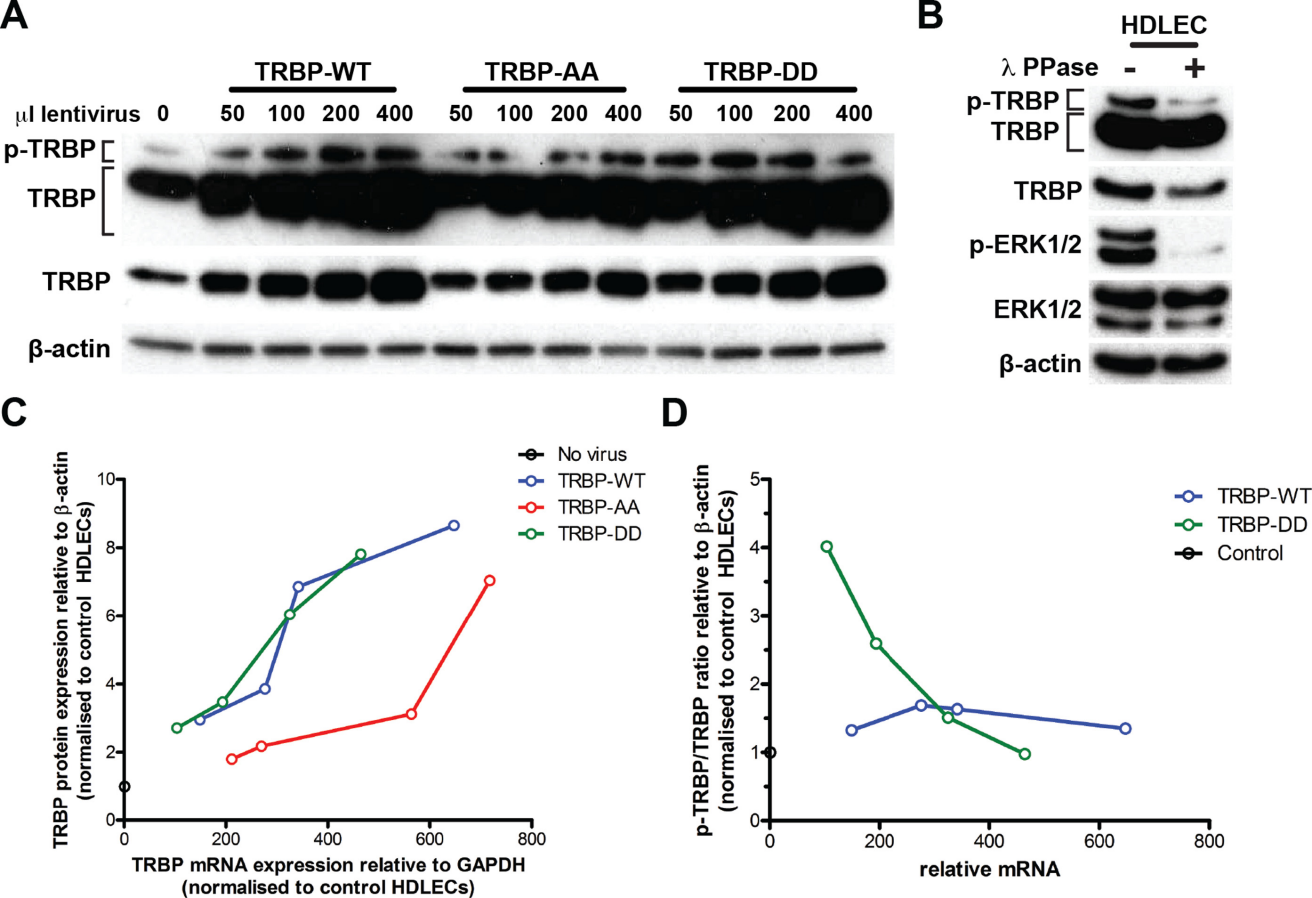
TRBP serines 283/286 control TRBP expression and post-translational modification. (**A**) Phosphorylated (p-TRBP) and total TRBP expression in HDLECs transduced (3 days, full media; see Materials and Methods) with the indicated volumes of lentiviruses (per 80 000 cells) encoding for TRBP-WT, TRBP-AA and TRBP-DD mutants. The top two rows of the immunoblots correspond to high (10–15 min) and low (1 min) exposure of the TRBP immunoblot, respectively. The band corresponding to hyper-phosphorylated TRBP is indicated as pTRBP. (**B**) Immunoblots showing TRBP and phosphorylated TRBP and phosphorylated ERK1/2 after lamda (λ) phosphatase treatment of HDLEC protein lysates. (**C**) Graph showing TRBP mRNA and protein levels for HDLECs transduced with 50, 100, 200, and 400 μl TRBP-WT, TRBP-AA, and TRBP-DD lentiviruses (per 80 000 cells, see Figure 7A). Levels are shown as fold induction compared to endogenous TRBP levels (no virus). (**D**) Graph showing the ratio of phosphorylated to total TRBP against TRBP mRNA levels for HDLECs transduced with TRBP-WT, and TRBP-DD lentiviruses. Levels are shown as fold induction compared to endogenous TRBP levels (no virus).

Next, we tested the functional relevance of serines 283/286 in cells. To provide physiological relevance of our findings, we utilized human primary cells rather than transformed cell lines, using primary HDLECs as a model system. We transduced HDLECs with lentiviral vectors encoding for full-length wild-type TRBP (TRBP-WT) or with mutants in which serines 283 and 286 have been mutated to alanines or aspartate (TRBP-AA or TRBP-DD, respectively). We titrated the three constructs into HDLECs and performed Western blot analyses (Figure 2A). The slower migrating TRBP band has been previously shown to correspond to hyper-phosphorylated TRBP (TRBP phosphorylated at multiple sites) (10,19). We confirmed that this band was sensitive to phosphatase treatment (from now on referred to as ‘phosphorylated TRBP’; p-TRBP in Figure 2B). During these titration experiments we ensured that TRBP mRNA levels were comparable between WT and TRBP mutants and found that TRBP expression in HDLECs was reduced when the two serines were mutated to alanines (Figure 2C) demonstrating that optimal TRBP expression and/or folding are directly linked to these two residues. Levels of phosphorylated TRBP increased following transduction of cells with increasing amounts of TRBP-WT, reaching a plateau for the highest amount of virus used (Figure 2A). When serines 283 and 286 were mutated to aspartate to mimic constitutive phosphorylation of these residues, there was an enhancement in p-TRBP/TRBP ratios at low lentivirus volumes (before the p-TRBP plateau was reached; Figure 2A and D; compare 50 and 100 μl virus lanes for TRBP-WT and TRBP-DD TRBP), but we did not observe an enhancement beyond that plateau. One possible explanation for this could be that when introducing high TRBP amounts in HDLECs, the abundance of hyperphosphorylated TRBP becomes limited by the amounts of kinases in these cells. Single mutation of serine 283 to alanine also negatively affected TRBP protein levels, whereas no differences were observed upon single mutation of serine 283 to aspartate (Supplementary Figure S1B and C).

**Figure 3.**
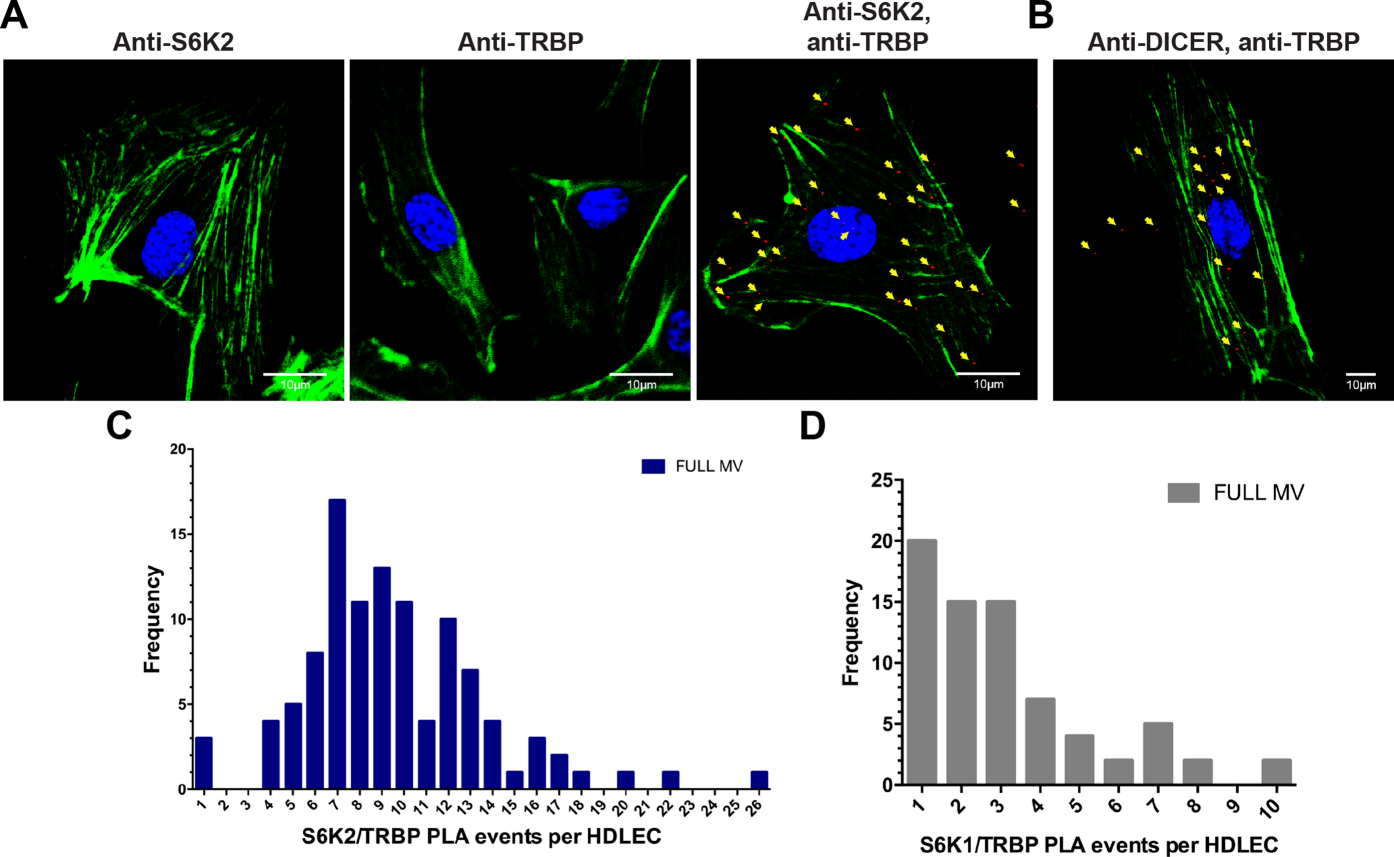
Endogenous TRBP interacts with S6Ks in primary HDLEC. (**A**) Analysis of TRBP and S6K2 interaction by PLA (red) in HDLECs growing in full growth factor media. Nuclei (blue) and F-actin (green) staining are also shown (scale bar: 10 μm). PLA events (in red) are indicated with yellow arrows. (**B**) Analysis of TRBP and DICER interaction by PLA (red) in HDLECs growing in full growth factor media. PLA events (in red) are indicated with yellow arrows. (**C**) Histogram of TRBP/S6K2 PLA events per cell detected in HDLECs cultured in full growth factor media. (**D**) Histogram of TRBP/S6K1 PLA events per cell detected in HDLECs cultured in full growth factor media.

Having shown that S6K2 and S6K1 can phosphorylate TRBP-D3 *in vitro* and that TRBP Ser283 and Ser286 contributed to optimal TRBP expression in primary cells, we investigated whether these kinases interact with TRBP in HDLECs. To determine potential endogenous interactions in primary cells, we used proximity ligation assays (PLAs) (29). We found that under optimal steady-state growth conditions (HDLECs growing in fully supplemented media—see Materials and Methods), endogenous S6K2 and TRBP directly interact *in situ* in HDLECs (Figure 3A, and Supplementary Figure S2). The TRBP/DICER interaction served as a positive control for the PLAs (Figure 3B). In addition to endogenous S6K2, our PLA analyses indicated that S6K1 can also interact with TRBP in HDLECs under steady state growth conditions (Figure 3C and D).

### S6K2 controls TRBP phosphorylation and expression in activated HDLECs

**Figure 4.**
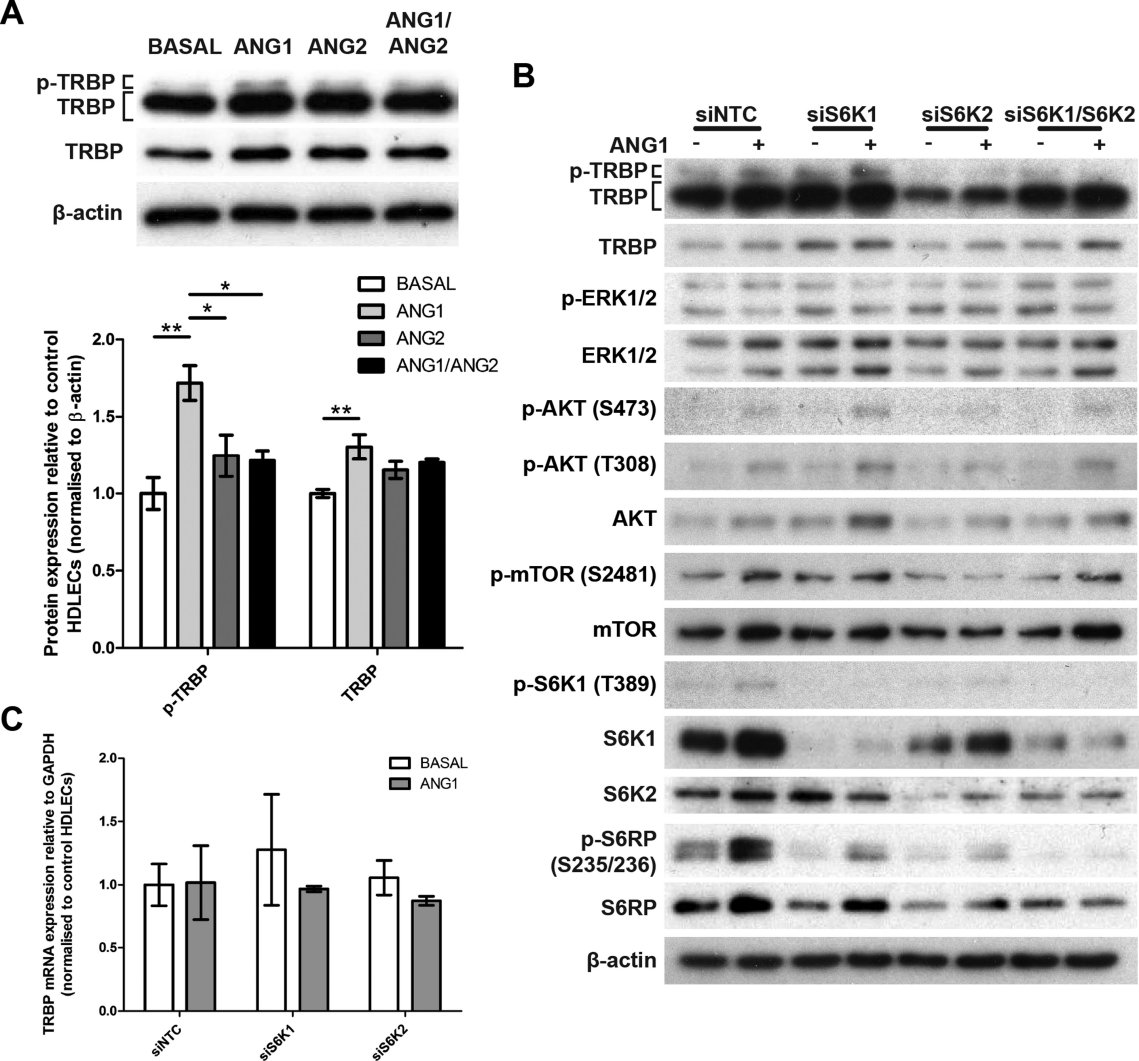
S6K2 is required for ANG1-mediated TRBP activation in HDLECs. (**A**) TRBP and p-TRBP levels in 24 h ANG stimulated HDLECs. Bar graph shows relative TRBP and p-TRBP levels in 24 h ANG stimulated HDLECs by densitometry. Values are normalized to cells grown in basal media (no growth factors) and are illustrated as mean ± SD (*n* = 3). *P* values are shown (*P < 0.05; **P < 0.01). (**B**) Levels of indicated total and phosphorylated proteins in control (–) and ANG1-stimulated (+) HDLECs (24 h) transfected with non-targeting control (NTC), S6K1, S6K2 and dual S6K1/S6K2 siRNAs. (**C**) TRBP mRNA levels in HDLECs transfected with non-targeting control (NTC), S6K1, S6K2, and grown without growth factors (Basal) or with ANG1 for 24 h.

To further investigate the significance of the TRBP/S6K2 interaction we exploited a well-characterized model of primary HDLEC activation and determined the effect of ANG1 and ANG2 on TRBP expression. Following incubation of subconfluent HDLEC cultures with ANG1 or ANG2, or both (18–24 h), we observed that ANG1 can induce a modest and reproducible increase in TRBP phosphorylation (Figure 4A, corresponding to the top band in TRBP immunoblots) and total TRBP expression (bottom band and lower exposure in TRBP immunoblots in Figure 4A). The ANG1 effect on TRBP phosphorylation was also confirmed using a different anti-TRBP antibody (Supplementary Figure S3A). These findings for endogenous TRBP were in agreement with biochemical and exogenous over-expression studies demonstrating that levels of TRBP phosphorylation control its stability and can correlate with total TRBP expression (10). The effect of ANG1 on hyperphosphorylated TRBP levels was reversed by ANG2, which is known to act as a TIE2 antagonist when added in endothelial cell cultures (20). ANG1-treated HDLECs demonstrated a statistically significant increase of AGO2 levels but no changes in levels of PACT, DICER, and DROSHA (Supplementary Figure S3B, S3C). ANG1 treatment did not affect TRBP mRNA levels, demonstrating that TRBP was not transcriptionally regulated under these conditions (Supplementary Figure S3D). TIE2 stimulation activates the ERK and AKT pathways (20) and the ERK pathway has already been linked to phosphorylation of TRBP serines 142 and 152 (10). In agreement with this, ANG1-induced phosphorylation of TRBP was dependent on TIE2 and ERK1/2 (Supplementary Figure S3E). The observed TIE2 downregulation in ANG1-treated cells is due to the previously reported activation-induced receptor internalization and degradation (30).

Next, we investigated whether S6K1 and S6K2 were also involved in ANG1 induced TRBP phosphorylation. By measuring levels of S6RP phosphorylation, a known substrate of S6 kinases, we confirmed that these kinases were active in HDLECs up to 24 h of ANG1 treatment (Figure 4B). As before, ANG1 treatment for 24 h resulted in increased TRBP phosphorylation. S6K1 knockdown enhanced TRBP phosphorylation and expression in both control and ANG1-treated cells. However, in S6K2 deficient HDLECs, the ANG1-induced TRBP phosphorylation and expression were impaired (Figure 4B and Supplementary Figure S4A). Of note, ANG1 did not induce TRBP phosphorylation in HDLECs lacking S6K1 and S6K2, with only minimal effects on total TRBP expression (Figure 4B, and Supplementary Figure S4A), demonstrating that S6K2 knockdown in S6K1-depleted cells specifically affected TRBP phosphorylation. Depletion of S6 kinases did not affect TRBP mRNA levels (Figure 4C). Both of these kinases are mTOR substrates and previously reported to be downstream of the AKT and ERK pathways, which are activated downstream of TIE2 (31,32). We observed mTOR and AKT activation after 24 h treatment of HDLECs with ANG1 (Figure 4B). ERK was not activated at this timepoint but we confirmed that it was activated at the early stages of ANG1 addition (Supplementary Figure S4B and (33)). Pre-treatment of HDLECs with Rapamycin resulted in reduction of TRBP phosphorylation and expression in ANG1-treated HDLECs (Supplementary Figure S4C), in agreement with mTOR controlling S6K activation. The effects of S6 kinases on TRBP were in agreement with S6K1, but not S6K2, mediating negative feedback loops affecting mTOR/AKT signalling (34,35). Indeed, we observed enhanced AKT activation in HDLECs lacking S6K1 (Figure 4B). Neither S6K2, nor S6K1 depletion affected phosphorylated ERK levels at 24 h (Figure 4B).

**Figure 5.**
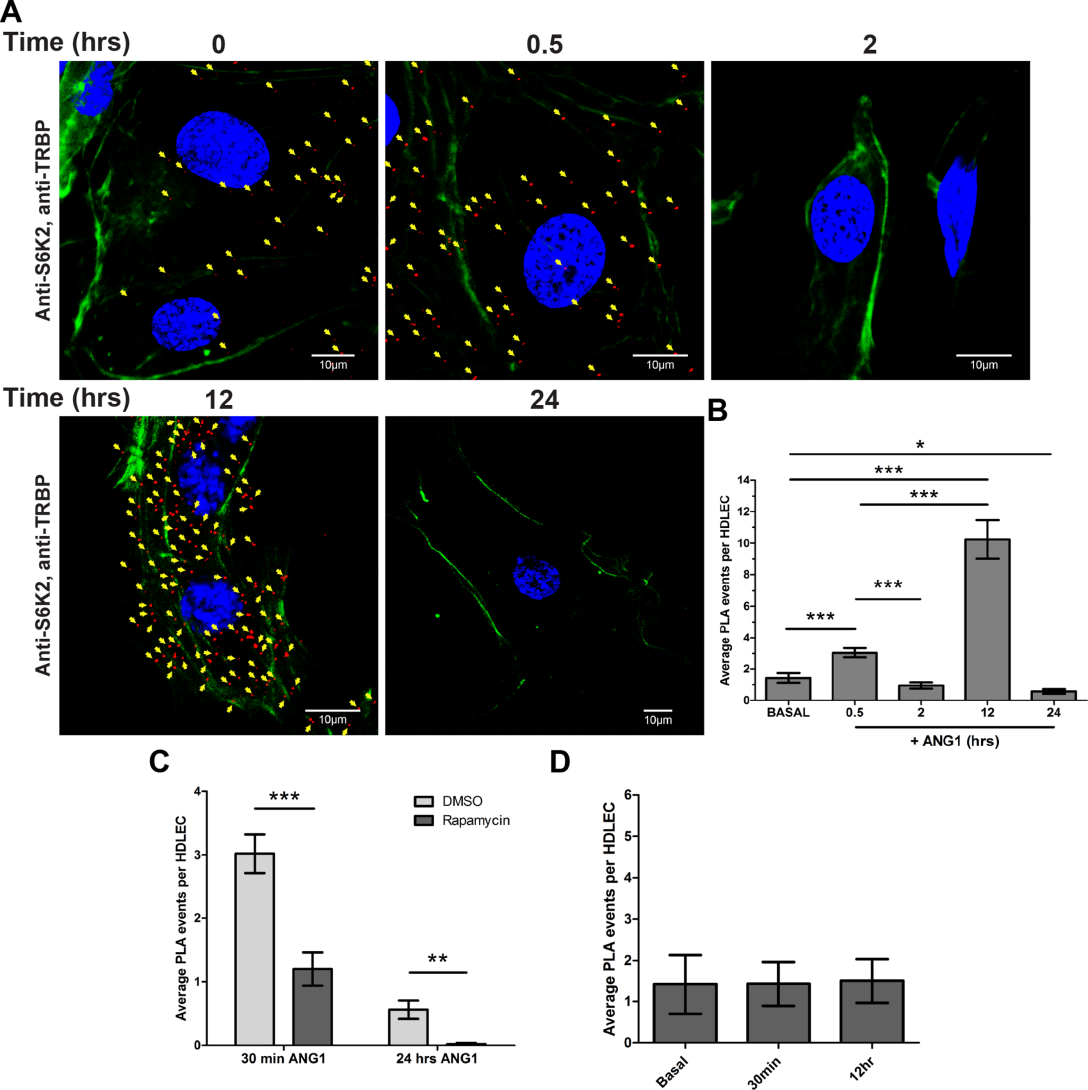
S6K2, but not S6K1, interacts with TRBP during ANG1 treatment of HDLECs. (**A**) Analysis of TRBP and S6K2 interaction by PLA (red) in HDLECs during 24 h ANG1 stimulation. Nuclei (blue) and F-actin (green) staining are presented in addition (scale bar: 10 μm). PLA events (in red) are indicated with yellow arrows. (**B**) Bar graph illustrating mean number of TRBP/S6K2 PLA events detected in HDLECs cultured in basal media or following overnight growth factor withdrawal and post 30 min, and 2, 12 and 24 h ANG1 stimulation. (**C**) Bar graph illustrating mean number of TRBP/S6K2 PLA events detected in HDLECs pre-treated with DMSO or Rapamycin (10 nM) for 2 h prior to 30min and 24 h ANG1 stimulation. (**D**) Bar graph illustrating mean number of TRBP/S6K1 PLA events detected in HDLECs cultured in Basal media or following overnight growth factor withdrawal and post 30 min, or 12 h ANG1 stimulation. Values presented are means ± SD. *P* values are shown (*P < 0.05; **P < 0.01; ***P < 0.001).

To further investigate the interaction of TRBP with S6Ks and dissect the differential effects of S6K1 and S6K2 on TRBP, we determined whether endogenous TRBP and S6Ks interact during ANG1 activation. We found that S6K2 and TRBP interacted in untreated HDLECs but the interaction was dramatically increased at 12 h post ANG1 addition (Figure 5A, B and Supplementary Figure S5A). There were no differences in TRBP phosphorylation and expression at 12hrs post ANG1 addition (Supplementary Figure S5B). The S6K2/TRBP interaction was also above background levels at 30 min following ANG1 addition (Figure 5A and B). Rapamycin pre-treatment significantly prevented the S6K2 and TRBP interaction (Figure 5C). Notably, the S6K1/TRBP interaction failed to exceed the levels observed in control cells during ANG1 treatment of HDLECs (Figure 5D) at time-points we observed maximal S6K2/TRBP interaction. This indicated that although both S6 kinases are capable of phosphorylating TRBP-D3 *in vitro* (Figure 1C), S6K2 is the predominant S6 kinase that associates with TRBP under these conditions.

**Figure 6.**
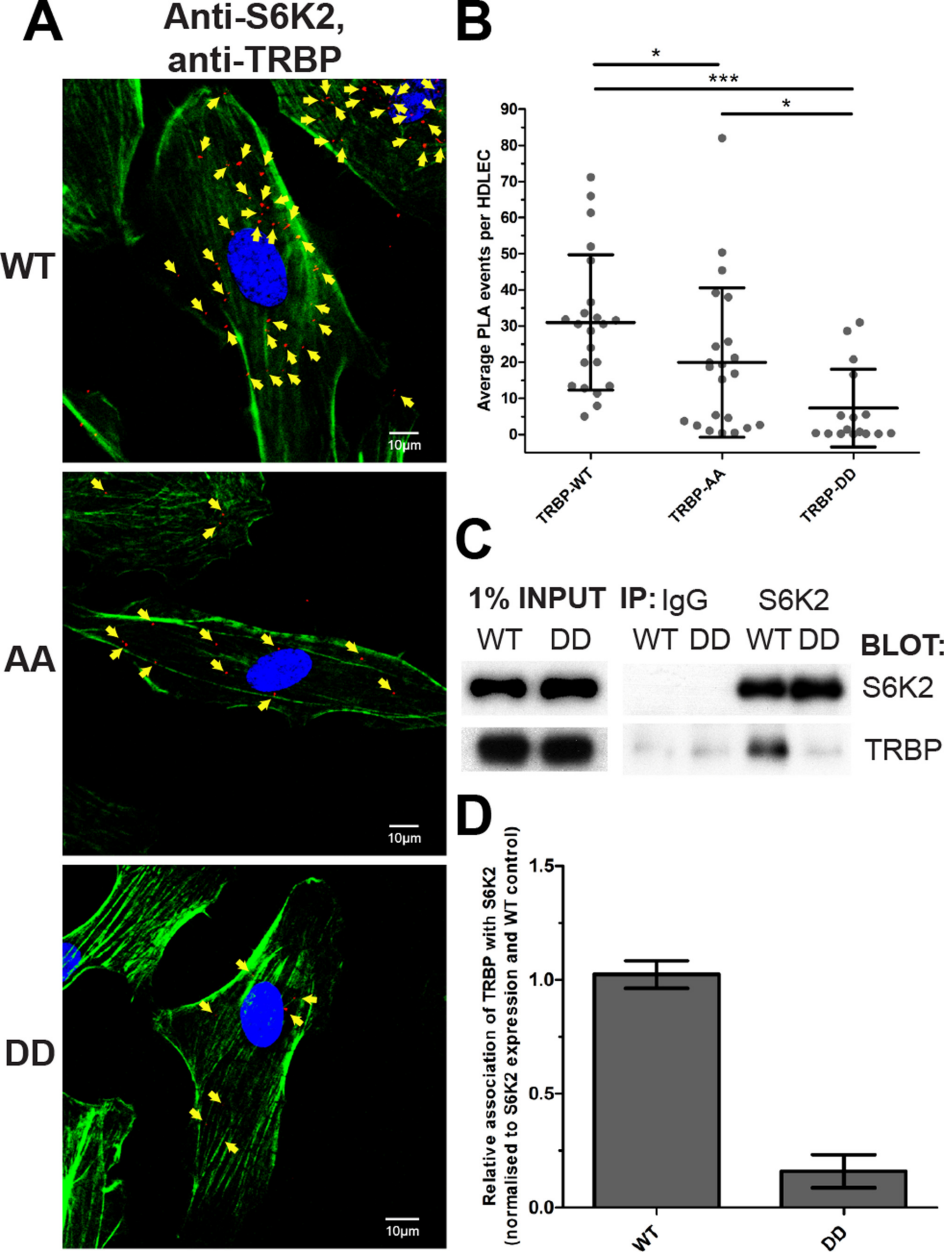
TRBP serines 283/286 are essential for the TRBP/S6K2 interaction in HDLECs. (**A**) PLA analysis of TRBP and S6K2 in HDLECs transduced with TRBP-WT (150 ml/80 000 cells), TRBP-AA (600 ml/80 000 cells), and TRBP-DD lentiviruses (200 ml/80 000 cells). The used volumes were calculated and verified to ensure equal TRBP protein expression between WT and mutant TRBP proteins. Nuclei (blue) and F-actin (green) staining are also shown (scale bar: 10 μm). PLA events (in red) are indicated with yellow arrows. B. Dot plot illustrating mean number of TRBP/S6K2 PLA events detected under the same conditions with (A). Values presented are means ± SD. *P* values are shown (*P < 0.05; ***P < 0.001). (**C**) Co-immunoprecipitation of S6K2 and TRBP in HEK293 cells over-expressing S6K2 and TRBP. Cells were transduced with S6K2 and TRBP-WT or TRBP-DD lentiviruses and harvested for immunoprecipitations when 80% confluent. (**D**) Quantification of co-immunoprecipitated TRBP with S6K2 in cells overexpressing TRBP-WT or TRBP-DD.

Having found that HDLEC activation resulted in increased association of S6K2, but not S6K1, with TRBP, we tested the effect of disrupting the AGC kinase-binding TRBP motif on the S6K2/TRBP interaction in cells. We transduced HDLECs with full-length TRBP-WT, TRBP-AA, and TRBP-DD lentiviruses, using appropriate volumes to achieve equal TRBP protein expression for all constructs. Using these cells, we found that the interaction of TRBP-WT with endogenous S6K2 was readily detectable. However, the TRBP/S6K2 PLA levels were significantly lower in HDLECs over-expressing TRBP-AA or TRBP-DD (Figure 6A, B and Supplementary Figure S6). The interaction between S6K2 and TRBP-DD was the weakest, supporting a model where TRBP phosphorylation at serines 283/286 promotes dissociation of TRBP from S6K2. This model was also consistent with the observation that maximum S6K2/TRBP interaction in ANG1-treated HDLECs (12hrs) preceded the peak of TRBP phosphorylation (24 h). Note that as over-expression experiments were performed in the presence of endogenous TRBP, some levels of S6K2/TRBP interaction were expected and observed in all conditions. To further validate these findings in a different cellular context, we overexpressed S6K2, TRBP-WT, and TRBP-DD in HEK293 cells and showed that TRBP-WT, but not TRBP-DD co-immunoprecipitates with S6K2 (Figure 6C and D). These experiments indicated that the S6K2/TRBP interaction is mediated through direct binding of the kinase to the TRBP phosphorylation motif (Figure 1A).

### TRBP and S6K2 contribute to enhanced miRNA expression in ANG1-treated HDLECs

**Figure 7.**
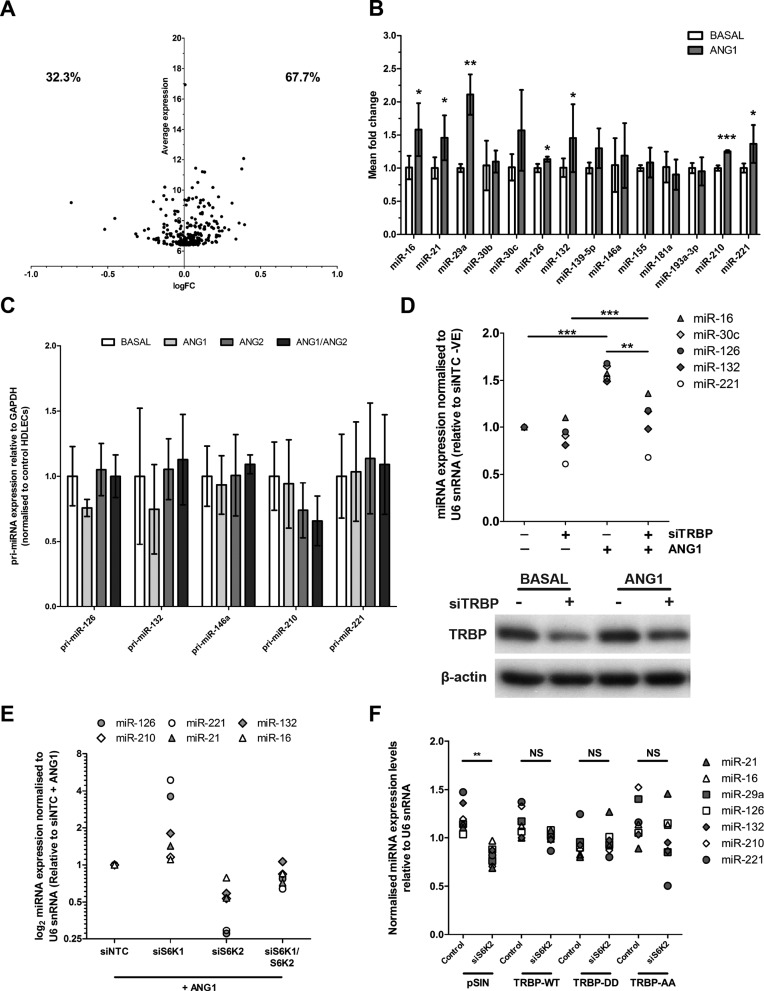
ANG1 enhances HDLEC miRNA expression in a TRBP- and S6K2-dependent manner. (**A**) Dot plot of miRNA log average expression against log fold change (FC) for all detectable miRNAs in HDLECs (average normalized signal ≥ 6.4). Percentages of down-regulated (negative log FC) and up-regulated (positive log FC) are shown. (**B**) Mature miRNA expression in control and 24 h ANG1 stimulated HDLECs determined by qRTPCR. Values are illustrated as mean ± SD (*n* = 4–6). (**C**) Primary miRNA transcript levels in 24 h Basal media, ANG1 and/or ANG2 treated HDLECs relative to cells grown in full growth factor containing media (*n* = 3). (**D**) Relative HDLEC miRNA expression in control and TRBP siRNA transfected cells post 24 h ANG1 stimulation or culture in Basal media. Immunoblot indicating knockdown levels also shown as insert. (**E**) miRNA expression in 24 h ANG1 stimulated HDLECs that had been transfected with S6K1 and/or S6K2 siRNAs. miRNA expression data shown is relative to control siRNA transfected HDLECs that had been stimulated for 24 h with ANG1. (**F**) miRNA expression in 24 h ANG1 stimulated HDLECs that had been transfected with control (NTC) or S6K2 siRNAs and lentiviruses encoding for TRBP-WT, TRBP-DD, and TRBP-AA (appropriate volumes of lentiviruses were used to achieve equal expression of WT and mutant TRBP). For each miRNA, expression levels shown are relative to the average expression of this miRNA amongst the eight tested samples shown in x-axis. Values presented are means ± SD. *P* values are shown (*P < 0.05; **P < 0.01; ***P < 0.001).

To determine the effect of ANG1 treatment on the HDLEC miRNA transcriptome, we performed miRNA profiling of control and ANG1-treated HDLECs (Supplementary Table S1). We observed that there was a statistically significant shift (*P* = 2.3 × 10^−5^) towards up-regulation for all detectable miRNAs (demonstrated as a positive log fold change for 276 miRNAs with average normalized signal >6.4; Figure 7A). We did not observe any expression shift when all miRNA probes present on the slides were included in the analysis confirming that signal normalization was robust (Supplementary Figure S7A). Notably, the proportion of miRNAs showing a positive fold change (FC) increased with increasing miRNA expression (Supplementary Figure S7A). To confirm that this was a true shift we tested the effect of ANG1 on expression of 14 mature miRNAs by qPCR. Seven of these miRNAs (miR-126, miR-21, miR-16, miR-29a, miR-221, miR-210 and miR-132) showed statistically significantly higher expression in ANG1-treated HDLECs (Figure 7B, and Supplementary Figure S7B). None of the tested miRNAs showed a statistically significant down-regulation upon ANG1 treatment. Furthermore, we confirmed that ANG1 had a significant effect on absolute copy number of two highly abundant miRNAs (miR-126 and miR-16; Supplementary Figure S7C, S7D). As in the case of TRBP phosphorylation (Figure 4A), the effect of ANG1 on miRNA expression was antagonized by concurrent addition of ANG2 (Supplementary Figure S7E). Notably, we did not observe any differences in levels of the primary transcripts of ANG1-regulated miRNAs (Figure 7C), indicating that ANG1 affected post-transcriptional miRNA regulation. These results demonstrated that ANG1 treatment had a widespread positive effect on miRNA expression in primary HDLECs and that for all the tested miRNAs this effect was post-transcriptional.

We then tested if the observed increase in TRBP phosphorylation and expression in ANG1-treated HDLECs (Figure 4A) contributes to the increased expression of ANG1-regulated miRNAs. As the effect of ANG1 on TRBP expression was modest we partially depleted TRBP in HDLECs and this ablated the effect of ANG1 on miRNA expression (Figure 7D). TRBP depletion also affected miRNA expression in HDLECs growing in full growth factor supplemented media and in cells growing in the basal media (absence of growth factors; Supplementary Figure S7F). Furthermore, we found that in HDLECs grown in basal media, TRBP overexpression was sufficient to increase miRNA expression (Supplementary Figure S7G). These findings indicated that under these conditions (growth factor starvation) TRBP levels become limiting, and increasing TRBP expression through overexpression (Supplementary Figure S7G) or ANG1 stimulation (Figure 4A) could result in increased miRNA expression.

Depletion of S6K2 resulted in a significant reduction in expression of miRNAs that were induced by ANG1 (miR-126, miR-16, miR-210, miR-21, miR-221 and miR-132, see Figure 7B) in ANG1-treated HDLECs (Figure 7E). In contrast, S6K1 depletion induced an increase in expression of the tested miRNAs, whereas levels of all tested miRNAs were reduced in S6K1/S6K2 depleted HDLECs in comparison to S6K1-depleted cells (Figure 7E). These results were consistent with the effects of S6K depletion on TRBP phosphorylation and expression (Figure 4B) and the effect of TRBP depletion on these miRNAs (Figure 7D). The statistically significant negative effect of S6K2 depletion on the expression of all tested miRNAs was not observed upon reintroduction of TRBP (Figure 7F), which at least partially rescued expression of all tested miRNAs. Exogenous expression of TRBP-AA or TRBP-DD (adjusted to achieve same TRBP expression levels) also partly rescued the effect of S6K2 depletion on miRNA expression. This suggested that, in ANG1-treated HDLECs, the predominant effect of S6K2-mediated TRBP phosphorylation is maintainance of optimal TRBP expression and miRNA abundance.

## DISCUSSION

Using human primary cells, we describe a previously uncharacterized S6K2-mediated mechanism controlling miRNA biogenesis in human primary cells, through regulation of TRBP phosphorylation and expression (Supplementary Figure S7H). Phosphorylation of TRBP-D3 by S6 kinases is a regulatory mechanism specific to TRBP and does not affect PACT, revealing a significant difference between the signal transduction mechanisms regulating the two DICER co-factors. In endothelial cells, this mechanism can be engaged through TIE2, a receptor tyrosine kinase with central roles in development, homeostasis, and pathological function of endothelium (20). This provides novel insight into how changes in the extracellular environment can affect intracellular miRNA biogenesis rates during blood and lymphatic vessel formation.

We demonstrate that growth factor-mediated TRBP activation can lead to significant and widespread changes in miRNA expression (Figure 7). The impact of ANG1 on HDLEC miRNA expression should be placed in the context of the described experimental conditions and previous reports demonstrating that ANG1 has anti-apoptotic and pro-proliferative effects on endothelial cells (36–38). Upon growth factor withdrawal, HDLECs are under apoptotic stress. ANG1 increases expression of some of the most highly expressed miRNAs in HDLECs (e.g. miR-126, miR-21, miR-16), for which a 1.2–1.5-fold enhancement corresponds to thousands of miRNA copies per cell. Concurring with previous reports proposing that the miRNA signature of a cell confers robustness during responses to stress (39) and that the miRNA-silencing machinery is required for endothelial cell survival (4–7), we speculate that the ANG1-mediated enhancement of miRNA expression might contribute to its pro-survival effects in endothelial cells.

The effects of ANG1 on HDLEC miRNA expression are achieved through regulation of TRBP phosphorylation and expression by S6 kinases. TRBP phosphorylation at 18 – 24h post ANG1 stimulation, is driven by S6 kinases and not ERK, which is not activated above background levels at these stages (Figure 4) but is still required for optimal TRBP activation (Supplementary Figure S3E). This suggests that ERK and S6 kinases collaborate to induce TRBP activation following ANG1 stimulation. Given the previously reported link between ERK and S6K activation (40), we should note that it is plausible that ERK can affect phosphorylation of TRBP indirectly through activating S6K2. Our findings are consistent with a phosphorylation-induced substrate dissociation model (41,42), suggesting that activated S6K2 phosphorylates TRBP, which then dissociates from S6K2 to bind DICER and enhance miRNA processing (Figures 1C, 5 and 6, Supplementary Figure S5).

We demonstrate that S6K1 and S6K2 play non-redundant and critical roles in the regulation of TRBP and miRNA expression in adult human endothelial cells. Although they share several common substrates and activities, non-redundant activities for the two S6 kinases have been previously reported (43,44). For example, S6K2, but not S6K1, controls cell proliferation in cancer cell lines (45,46). The differential effects of the two kinases in the context of TRBP activation in ANG1-treated HDLECs (Figure 4B) are due to the fact that the two kinases participate in distinct feedback regulatory loops (35), and can display distinct subcellular localization patterns (47). Indeed, during ANG1 treatment of HDLECs, TRBP is found in the proximity of S6K2 at significantly higher levels than those observed in control cells, however this is not the case for S6K1 and TRBP (Figure 5). Furthermore, depletion of S6K1 results in compensatory activation of signalling pathways (e.g. increased AKT activation; Figure 4B), while S6K2 depeltion does not have this effect. However, as TRBP-D3 can be phosphorylated by both kinases *in vitro* (Figure 1C), our results do not exclude the possibility that TRBP phosphorylation can be predominantly S6K1-dependent in other growth conditions or cellular contexts.

Implicating S6 kinases in the regulation of miRNA biogenesis can have far-reaching implications. We speculate that regulation of mature miRNA levels through mTOR/S6K-mediated TRBP regulation is likely to be involved in other, non-endothelial, contexts of cellular activation. Through identifying TRBP as a novel S6K substrate, we provide a mechanism that links the mTOR pathway to miRNA biogenesis. We speculate that this is a fundamental mechanism co-ordinating mRNA translation and RNA-mediated silencing in mammalian cells.

## Supplementary Material

SUPPLEMENTARY DATA
