# Supplementary material for: S6K2-mediated regulation of TRBP as a determinant of miRNA expression in human primary lymphatic endothelial cells
Source: Nucleic Acids Res. 2016 Jul 12;44(20):9942–55. doi: 10.1093/nar/gkw631 (PMC5175334; doi:10.1093/nar/gkw631)
Supplement: SUPPLEMENTARY DATA [file supp_44_20_9942__index.html]

S6K2-mediated regulation of TRBP as a determinant of miRNA expression in human primary lymphatic endothelial cells — SUPPLEMENTARY DATA 

# S6K2-mediated regulation of TRBP as a determinant of miRNA expression in human primary lymphatic endothelial cells

## SUPPLEMENTARY DATA

- SUPPLEMENTARY DATA
- SUPPLEMENTARY DATA
- SUPPLEMENTARY DATA
- SUPPLEMENTARY DATA
- SUPPLEMENTARY DATA
- SUPPLEMENTARY DATA
- SUPPLEMENTARY DATA
- SUPPLEMENTARY DATA
- SUPPLEMENTARY DATA
