# Supplementary figures and images for: S6K2-mediated regulation of TRBP as a determinant of miRNA expression in human primary lymphatic endothelial cells
Source: Nucleic Acids Res. 2016 Jul 12;44(20):9942–55. doi: 10.1093/nar/gkw631 (PMC5175334; doi:10.1093/nar/gkw631)

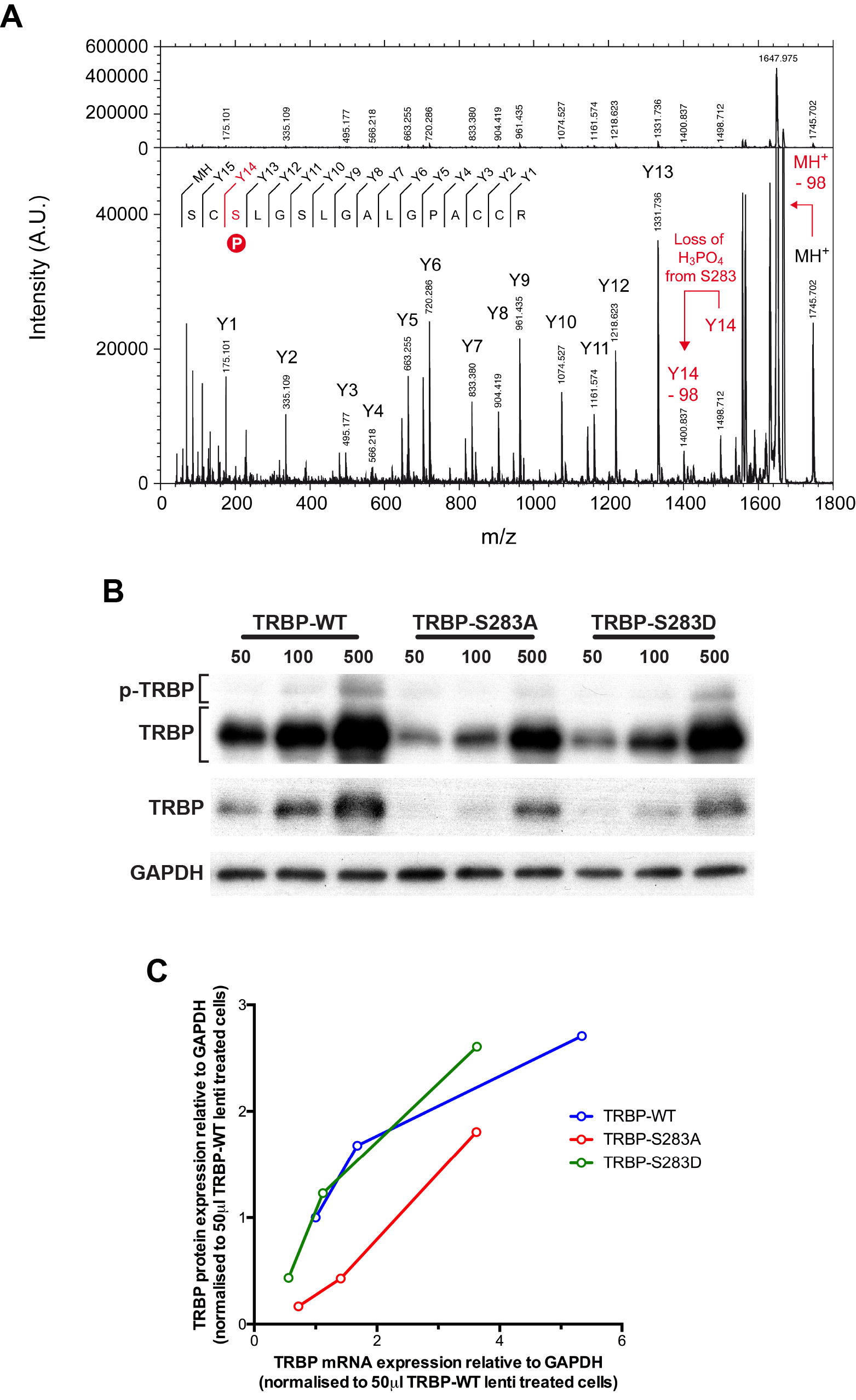

Supplement: SUPPLEMENTARY DATA [file supp_gkw631_Figure_S1.tif]

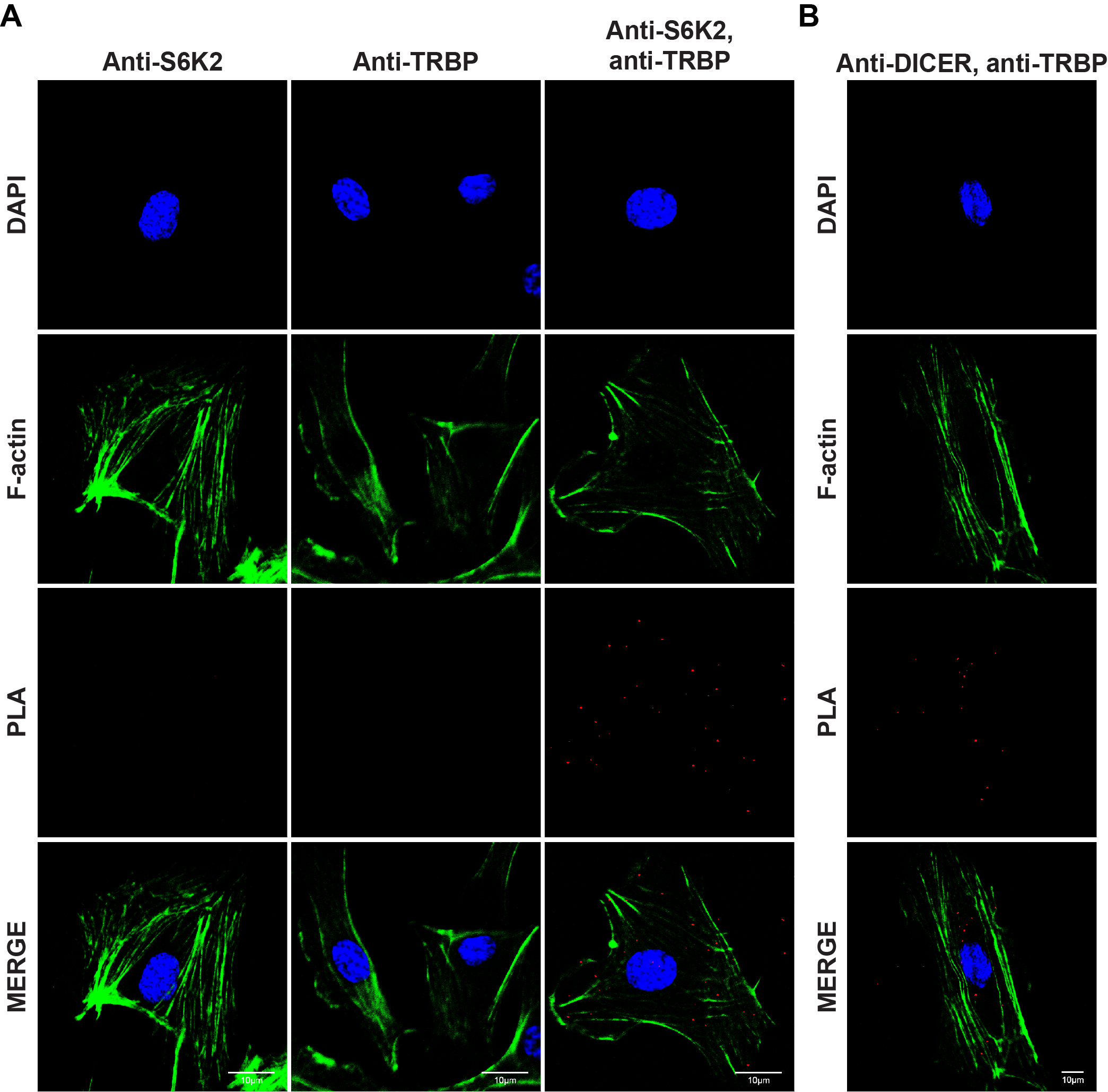

Supplement: SUPPLEMENTARY DATA [file supp_gkw631_Figure_S2.tif]

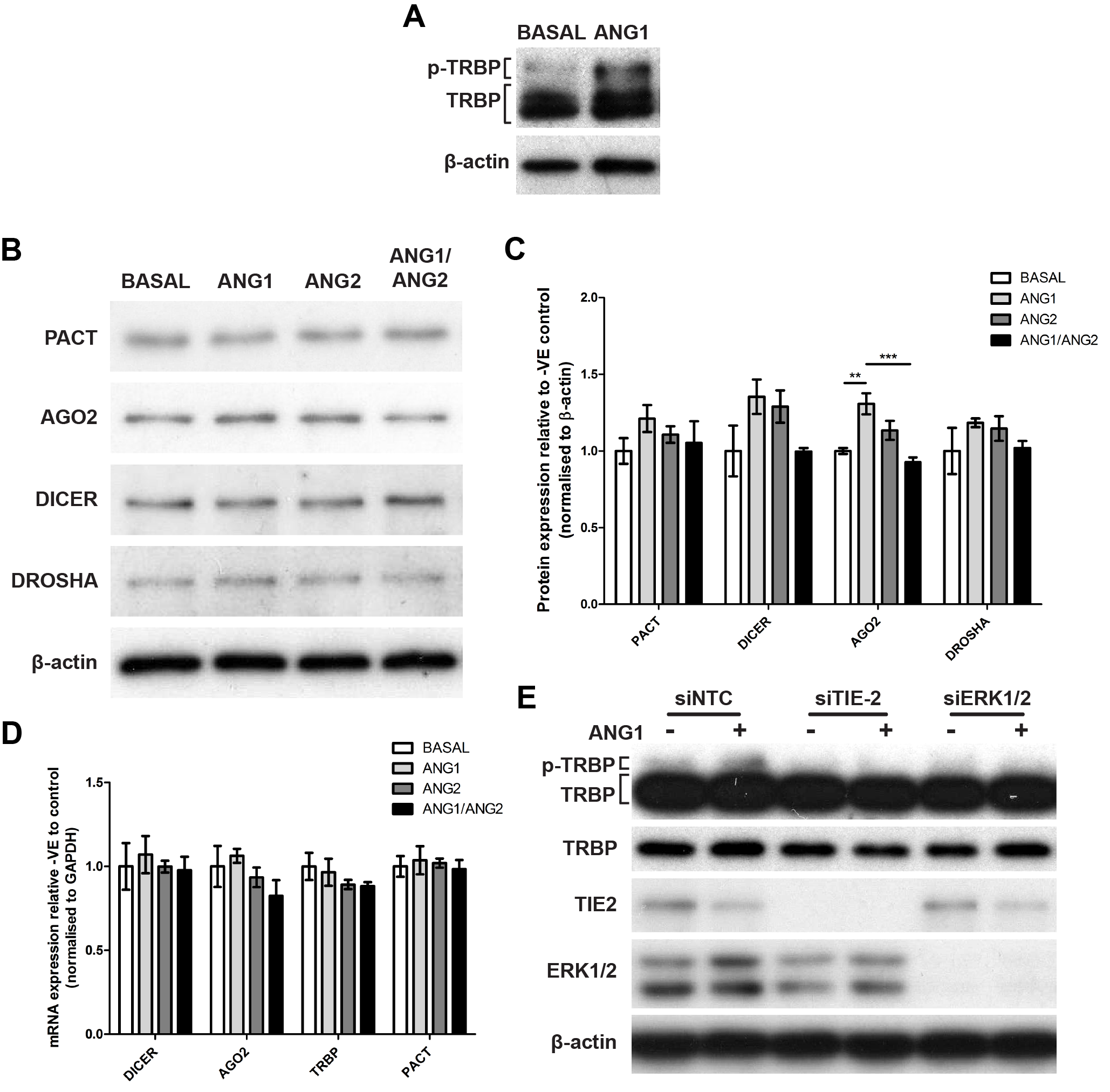

Supplement: SUPPLEMENTARY DATA [file supp_gkw631_Figure_S3.tif]

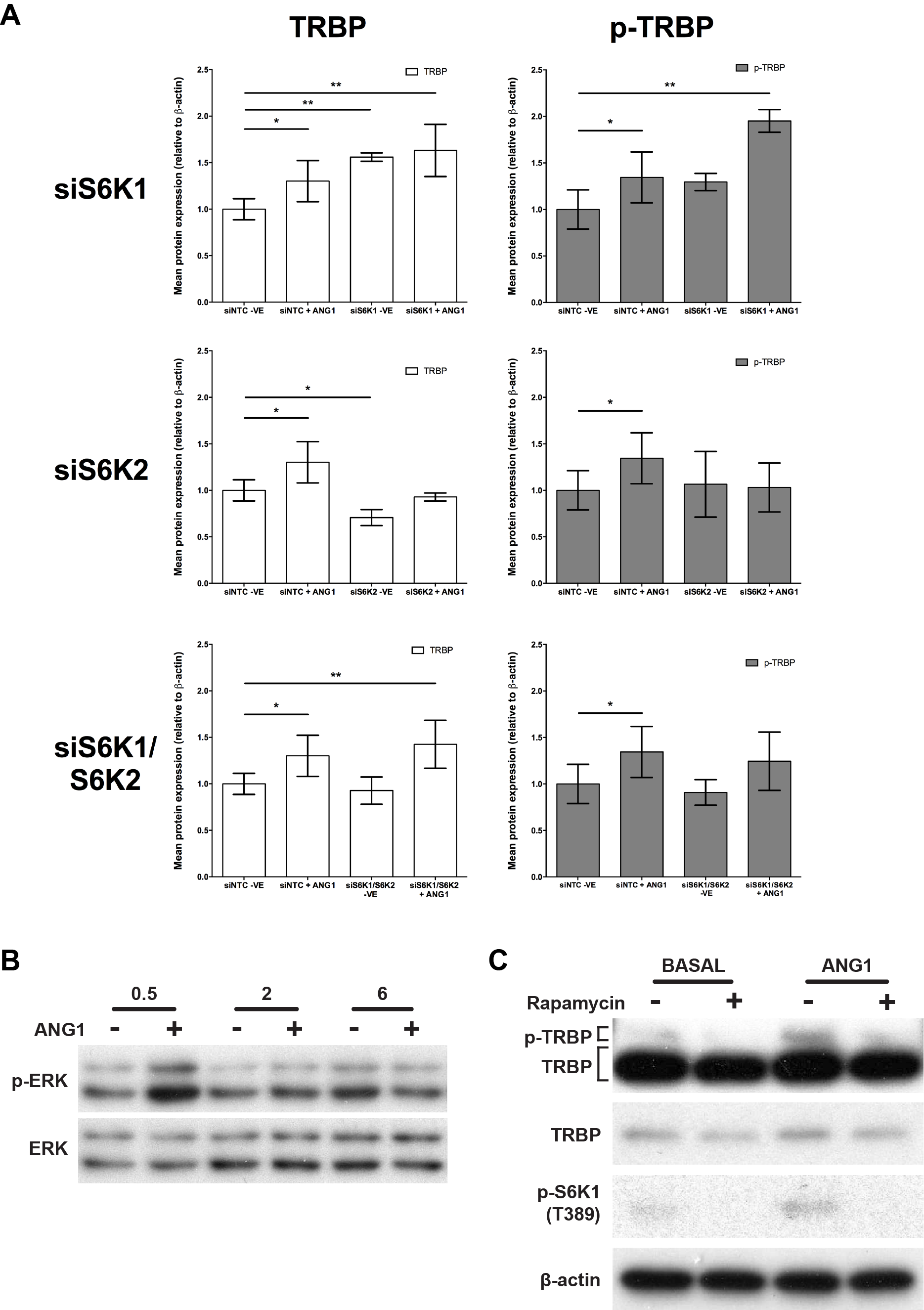

Supplement: SUPPLEMENTARY DATA [file supp_gkw631_Figure_S4.tif]

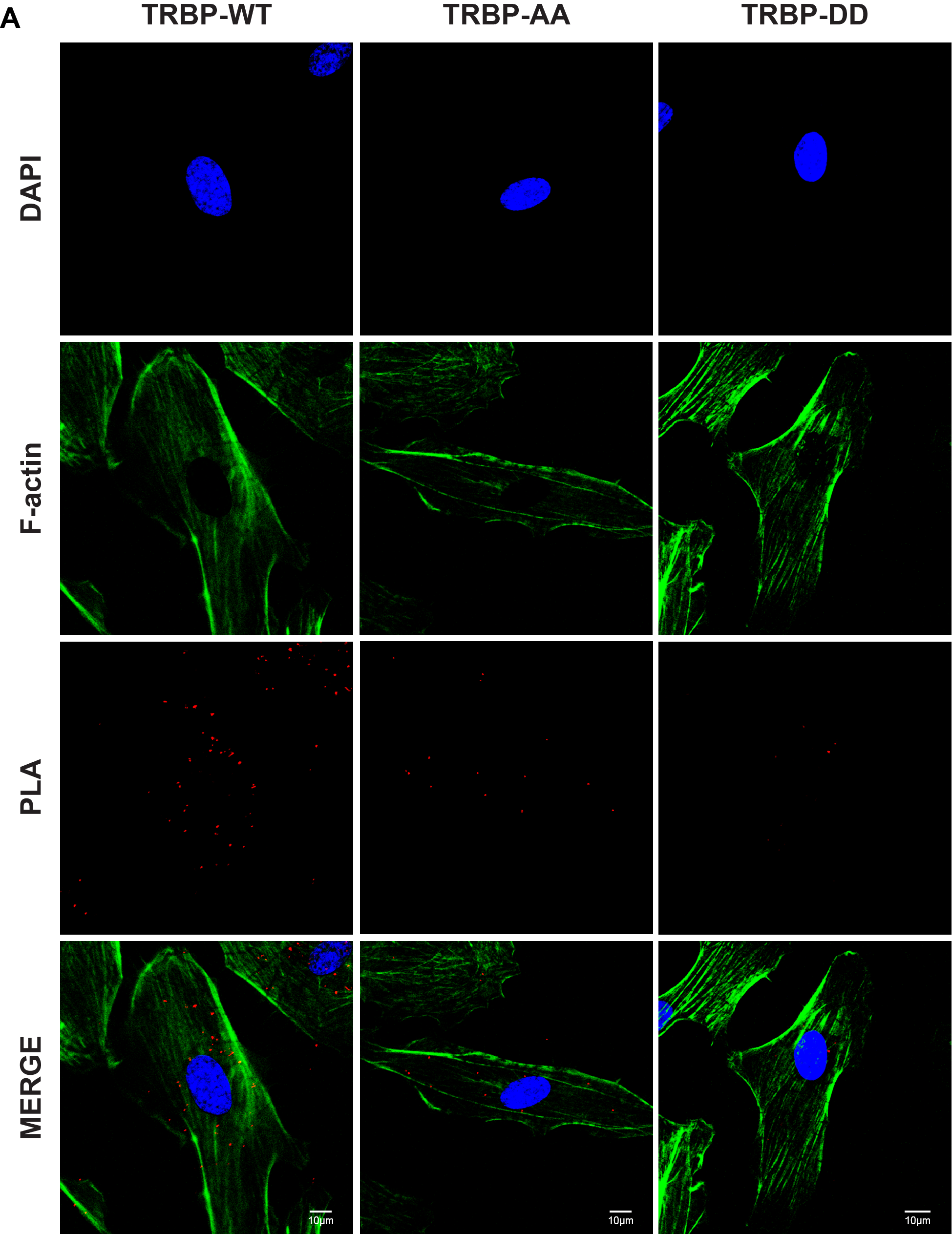

Supplement: SUPPLEMENTARY DATA [file supp_gkw631_Figure_S6.tif]

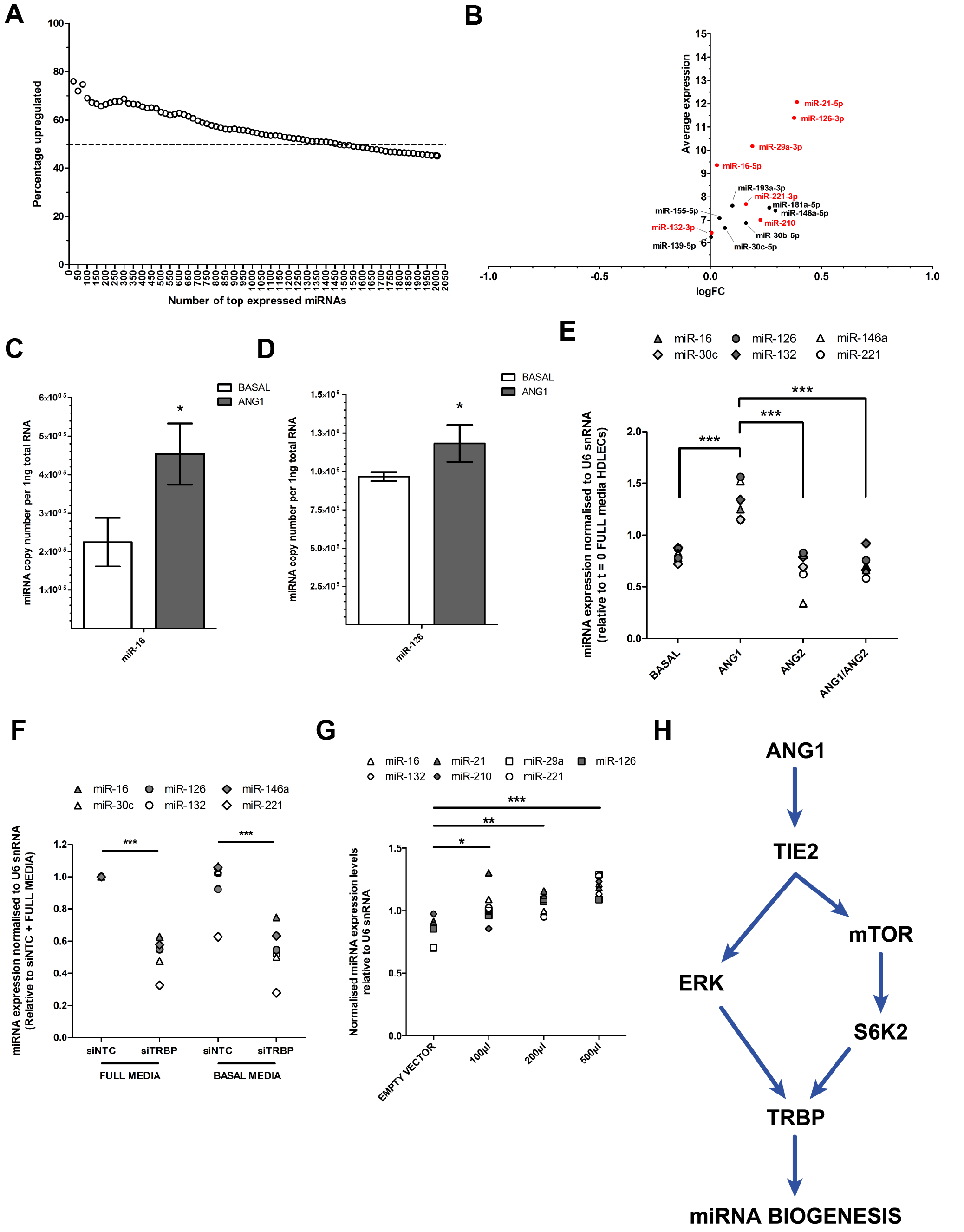

Supplement: SUPPLEMENTARY DATA [file supp_gkw631_Figure_S7.tif]
