# Supplementary material for: S6K2-mediated regulation of TRBP as a determinant of miRNA expression in human primary lymphatic endothelial cells
Source: Nucleic Acids Res. 2016 Jul 12;44(20):9942–55. doi: 10.1093/nar/gkw631 (PMC5175334; doi:10.1093/nar/gkw631)
Supplement: SUPPLEMENTARY DATA [file supp_gkw631_nar-01681-v-2016-File009.pdf]

Supplementary Figure 1 – AGC kinases phosphorylate TRBP and affect its expression in primary HDLECs

A. MS/MS fragmentation of a tryptic peptide of TRBP-D3b m/z 1745, following *in vitro* kinase activity assay of S6K2 and TRBP-D3. Top panel, full MS/MS spectrum. Bottom panel, zoom to highlight lower intensity peaks. Peaks of interest are annotated to indicate Y ions and m/z values. Y14 (corresponding to serine 283) and the parent ion peaks show an associated peak that corresponds to a loss of 98 Da, highlighted in red.

B. Phosphorylated (p-TRBP) and total TRBP expression in HDLECs transduced (3 days, full media) with the indicated volumes of lentiviruses (per 80,000 cells) encoding for TRBP-WT, TRBP-A, and TRBP-D mutants (mutated at serine 283).

C. Graph showing TRBP mRNA and protein levels for HDLECs transduced with 50, 100, 200, and 400 μl TRBP-WT, TRBP-A, and TRBP-D lentiviruses (per 80,000 cells, as in Supplementary Figure S1A). Levels are shown as fold induction compared to HDLECs transduced with 50 μl TRBP-WT lentivirus.

Supplementary Figure 2 - Endogenous TRBP interacts with S6Ks in primary HDLEC

A. Deconvolution of Figure 3A. Analysis of TRBP and S6K2 interaction by PLA (red) in HDLECs growing in full growth factor media. Nuclei (blue) and F-actin (green) staining are also shown (scale bar: 10 μm).

B. Deconvolution of Figure 3B. Analysis of TRBP and DICER interaction by PLA (red) in HDLECs growing in full growth factor media.

Supplementary Figure 3 – ANG1 enhances TRBP phosphorylation and expression in HDLECs

A. Phosphorylated (p-TRBP) and total TRBP expression in HDLECs grown in basal media or treated with ANG1 (24 hrs), as assessed with anti-TRBP antibody 15753-1-AP (Proteintech).

B. Immunoblots of miRNA biogenesis components in 24 h ANG stimulated HDLECs.

C. Densitometry analysis for miRNA biogenesis factors in 24 h ANG treated HDLECs. Values are presented as means ± SD (n=3). *P* values are shown (** = $p < 0.01$; *** = $p < 0.001$).

D. mRNA expression of miRNA biogenesis factors in 24 h ANG treated HDLECs. Values are presented as means ± SD (n=3).

E. Immunoblots of TIE2, ERK1/2, and TRBP in 24 h basal and ANG1-stimulated HDLECs transfected with control, TIE2 and ERK1/2 siRNA.

Supplementary Figure 4 – TRBP expression in ANG1-treated HDLECs is S6K2-dependent

A. Total and phosphorylated TRBP densitometry data in basal media (indicated as "-ve") and 24 h ANG1-stimulated HDLECs after cells had been transfected with non-targeting control (siNTC), S6K1, S6K2 and dual S6K1/S6K2 siRNAs (n=3). Data is presented as means ± SD relative to control siRNA transfected HDLECs treated for 24 h in basal media. *P* values against the "siNTC/-ve" samples are shown (* = $p < 0.05$; ** = $p < 0.01$; *** = $p < 0.001$).

B. Levels of ERK1/2 and phosphorylated ERK1/2 at the indicated timepoints following addition of ANG1.

C. Levels of total and phosphorylated TRBP and phosphorylated S6K1 (T389) in HDLECs pre-treated with DMSO, or rapamycin (10 nM) for 2 h prior to 24 h ANG1 stimulation.

Supplementary Figure 5 – TRBP interacts with S6K2 during ANG1-mediated activation of HDLECs

A. Deconvolution of Figure 5A. Analysis of TRBP and S6K2 interaction by PLA (red) in HDLECs during 24 h ANG1 stimulation. PLA (red, Nuclei (blue) and F-actin (green) staining are shown (scale bar: 10 µm).

B. TRBP levels in HDLECs grown in basal media or treated with ANG1 for 12 hrs.

Supplementary Figure 6 – The AGC-binding TRBP motif is necessary for the TRBP/S6K2 interaction in HDLECs

Deconvolution of Figure 6A. PLA (red), nuclei (blue) and F-actin (green) are shown (scale bar: 10 µm).

Supplementary Figure 7 – TRBP mediates the ANG1-induced enhancement in HDLEC miRNA expression

A. Percentage of miRNAs demonstrating a positive log fold change (FC) in ANG1-treated HDLECs compared to control HDLECs (Y-axis). The X-axis indicates the number of miRNAs included in the analysis ranked by expression. For example nearly 80% of the top 50 expressed miRNAs in HDLECs show a positive log FC in HDLECs, whereas when the analysing all 2006 miRNAs measured in the array only approximately 45% show a positive log FC.

B. Scatter plot illustrating average expression (normalised signal) and

log FC between control and ANG1-stimulated HDLECs (24h) for miRNAs tested by qRT-PCR (see Fig. 1C). miRNAs that showed significant up-regulation in Fig. 7B are shown in red.

C. Absolute miR-16 copy number per ng of total RNA in ANG1-treated and negative control (-VE) HDLECs

D. Absolute miR-126 copy number per ng of total RNA in ANG1-treated and negative control (-VE) HDLECs

E. miRNA expression in HDLECs grown in Basal, ANG1-, ANG2-, or ANG1/ANG2-supplemented media for 24h, following 16h starvation. Levels are relative to cells grown in full growth factor supplemented media.

F. Relative HDLEC miRNA expression in control and TRBP siRNA transfected cells grown in full growth factor supplemented or basal media.

G. Relative HDLEC miRNA expression in HDLECs transduced with control lentivirus (Empty vector) or increasing amounts of TRBP-WT lentivirus (100μl, 200μl, or 500μl per 80,000 cells). HDLECs were transduced and, 48 hrs later, the media changed to Basal media. RNA was collected 24 hrs later. For each miRNA, expression levels shown are relative to the average expression of this miRNA amongst the eight tested samples shown in x-axis. For levels of TRBP expression corresponding to indicated amounts of virus see Figure 2A.

H. Schematic summary of the ANG1/mTOR/S6K2/TRBP-mediated enhancement of miRNA biogenesis in HDLECs.

P values are shown throughout (* = $p < 0.05$; ** = $p < 0.01$; *** = $p < 0.001$).

Supplementary Table 1 – MiRNA profiling analysis using Agilent miRNA Chips

Normalised average signal, log fold change, and statistical analysis per miRNA in

ANG1-treated HDLECs at 24h compared to control HDLECs.
